# Coarctation of the Aorta: Diagnosis and Management

**DOI:** 10.3390/diagnostics13132189

**Published:** 2023-06-27

**Authors:** Sadaf Raza, Suneil Aggarwal, Petra Jenkins, Ahmed Kharabish, Shehab Anwer, Damien Cullington, Julia Jones, Jaspal Dua, Vasileios Papaioannou, Reza Ashrafi, Sarah Moharem-Elgamal

**Affiliations:** 1 Adult Congenital Heart Disease Centre, Liverpool Heart and Chest Hospital, Liverpool L14 3PE, UK; 2 Radiology Department, Liverpool Heart and Chest Hospital, Liverpool L14 3PE, UK; 3 Radiology Department, Al Kasr Al Aini, Old Cairo, Cairo 11562, Egypt; 4 Cardiology Department, University of Zurich, 8006 Zurich, Switzerland; 5 Cardiology Department, National Heart Institute, Giza 11111, Egypt

**Keywords:** coarctation of the aorta, congenital heart disease, diagnostic imaging, intervention, surgery

## Abstract

Coarctation of the aorta (CoA) accounts for approximately 5–8% of all congenital heart defects. Depending on the severity of the CoA and the presence of associated cardiac lesions, the clinical presentation and age vary. Developments in diagnosis and management have improved outcomes in this patient population. Even after timely repair, it is important to regularly screen for hypertension. Patients with CoA require lifelong follow-up with a congenital heart disease specialist as these patients may develop recoarctation and complications at the repair site and remain at enhanced cardiovascular risk throughout their lifetime.

## 1. Introduction

Coarctation of the aorta (CoA) is one of the most common congenital heart defects (CHD), accounting for approximately 5–8% of CHD [1], with an incidence of 4 per 10,000 live births [2]. Clinical presentation can vary depending on a number of factors including the severity of the CoA and the presence of associated cardiac lesions, particularly those relating to left-sided heart obstruction. In the presence of severe CoA, neonates may present with cardiovascular collapse, particularly at the time of ductal closure. At the other end of the spectrum, CoA may be diagnosed in adult patients who may present with complications of long-standing secondary hypertension.

There have been significant advances in the diagnosis and management of CoA with the advent of multi-modality imaging, rapid advances in interventional techniques and the accumulation of long-term data for patients who have been operated on in childhood. The development of coordinated care for patients with adult congenital heart disease in the developed world has also led to improvements in outcomes. There is increasing evidence that CoA represents a generalised arteriopathy, leading to an enhanced cardiovascular risk across the patient’s lifetime and the need for lifelong follow-up with an adult congenital heart disease specialist.

## 2. History and Anatomical Descriptions

The anatomist Morgagni described CoA of the aorta in 1760, with the first operation being performed by the Swedish surgeon Clarence Crafoord in 1944, who completed an end-to-end anastomosis of the aorta on two patients aged 12 and 27 years [3]. CoA represents a spectrum of anatomical variants of the aorta, usually in the region of the ductus arteriosus, ranging from a discrete narrowing within the aortic arch to tubular hypoplasia of the aorta and aortic atresia [4]. Anatomical classification of CoA has traditionally relied on the relationship of the point of obstruction to the ductus arteriosus, with juxta-ductal stenosis being the case in the majority, although pre-ductal and post-ductal stenosis may also be seen. A discrete shelf-like lesion located posteriorly within the aorta is the most common anatomical variant seen.

Beyond a purely anatomical description, however, the definition of CoA within international guidelines relies on haemodynamic data from cardiac catheterisation [5,6]. For example, the ESC guidelines for adult congenital heart disease state, “Cardiac catheterization with manometry (a peak-to-peak gradient ≥ 20 mmHg) indicates a haemodynamically significant CoA, in the absence of well-developed collaterals.” 

However, the haemodynamic definition of CoA may have significant limitations. This was highlighted in recent work modelling fluid dynamics in CoA, which demonstrated the dependence of the trans-stenotic gradient on systemic compliance [7]. Systemic arterial compliance is not accounted for in current haemodynamic definitions of significant CoA and is often low in these patients, who have a high prevalence of hypertension. Reduced arterial compliance leads to a reduction in the observed gradient and therefore potential under-estimation of the severity of the obstruction. The clinical diagnosis of CoA, therefore, relies on a constellation of supportive clinical data points including the presence of anatomical obstruction as demonstrated on cross-sectioning imaging. This, together with echocardiography-based Doppler gradients, cardiac catheter data, as well as physical examination findings such as discrepant upper and lower limb blood pressures, help to define the presence of significant CoA or recurrent CoA.

CoA is associated with several cardiac lesions, with associated congenital cardiac anomalies being reported in a majority of patients with CoA in recent case series [8,9]. Bicuspid aortic valve has been reported to be present to a variable degree in between 25 and 85% of patients with CoA [10]. This association is important since bicuspid aortic valve is significantly associated with a distinct aortopathy, characterised by progressive aortic root dilatation, with a higher potential for an aortic complication in these patients in the long term. Other cardiac associations include those causing increased pulmonary flow, such as perimembranous VSD and double outlet right ventricle, and left-sided obstructive lesions such as supra or subvalvular aortic stenosis, congenital mitral stenosis and hypoplastic left heart syndrome [11]. Shone’s syndrome is the association of parachute mitral valve, supravalvar left atrial ring, subaortic stenosis and CoA [11,12]. Non-cardiac associations include Berry aneurysms, present in 10% of patients [13]. Anomalies of the coronary and renal arteries may also be seen. 

## 3. Pathophysiology and Genetics

The genetic basis of CoA is poorly understood, although there is a significant risk of familial recurrence suggesting a heritable component [14]. Candidate genes for non-syndromic inheritance include NOTCH1, MCTP2 and MATR-3. [15] NOTCH-1 is also associated with familial bicuspid aortic valve disease [16]. A familial case of bicuspid aortic valve and CoA has been described in association with KCNJ2 mutation, which is more commonly associated with arrythmias in those with Andersen–Tawil syndrome [17]. The most common syndromic association is with Turner’s syndrome (45X), with CoA present in approximately 10% and less common in those with mosaic monosomy [18,19].

There are a number of theories regarding the pathophysiology of CoA, which can be summarised as follows:

1. Extension of ductal tissue into the aorta, causing constriction of the aorta when the ductus arteriosus closes at birth
2. Hemodynamic reduction in LV forward flow leading to abnormal development of the aorta, which would be supported by the association of other left-sided obstructive lesions with CoA
3. Abnormalities of the migration of neural crest cells which are the origin of parts of the aorta and left ventricular outflow tract and valves [20].

The most studied of these theories is the ductal theory, with substantial evidence in the literature for the histological and biomolecular similarity of ductal and CoA tissue.

Histologically, normally developed ductal tissue demonstrates loosely arranged smooth muscle cells without elastic fibres. Fetal ductal arteriosus specimens have been shown to display advanced smooth muscle cell differentiation compared to adjacent aortic tissue in a number of studies, with electron microscopy revealing the presence of well-developed contractile myofilaments in ductal tissue which is thought to be an important contributor to ductal closure soon after birth [21,22]. Dedifferentiated SMCs have also been demonstrated in ductal tissue in neonatal specimens, with evidence of cystic necrosis and apoptotic cells in the tunica media. 

Elzenga and Gittenberger-de Groot et al. studied 45 CoA specimens obtained at surgery. CoA specimens were characterised by the presence of intimal thickening, with ductal tissue forming half of the total circumference of the CoA segments in comparison to normal aortas where it formed less than one-third of the circumference [23]. CoA segments share similarities with ductal tissue on histology. They are typically characterised by intimal thickening with an expansion of the extracellular matrix. Smooth muscle cells (SMCs) are seen with fragmentation of the elastic lamina, a feature which is also seen in ductal tissue. Smooth muscle cells within the intima of CoA segments demonstrate a mixture of differentiated and dedifferentiated phenotypes, with more differentiated SMCs being found in the outer intimal and juxtamedial position [24]. The origin of these smooth muscle cells remains unclear. Recent work points to a synthetic phenotype of SMCs in the coarctation shelf with a fibroblast-like morphology [25]. The pattern of differentiated SMCs and dedifferentiated SMCs in the intimal and medial layers has similarities to the histology of the normally developed ductus arteriosus tissue, lending support to the ductal theory. Apoptotic cells have also been demonstrated in resected CoA tissue with evidence of cystic medial necrosis. Normal ductal tissue demonstrates the presence of apoptotic cells, with apoptosis being an important mechanism in the remodelling process which leads to the obliteration of the ductus arteriosus.

A deeper understanding of the cellular mechanism of CoA is important in guiding therapeutic approach. Given the presence of apoptotic cells in the CoA segment, there may be a higher risk of aortic aneurysm when surgical techniques are employed that leaves abnormal ductal tissue intact within the aorta such as subclavian flap repair and patch aortoplasy, when compared to end-to-end anastomosis of the aorta, which completely resects the abnormal tissue [26].

The ductal theory does not however explain the pathophysiology of CoA in its entirety though, since the coarctation site can be at some distance from the site of the ductus arteriosus. Since the vascular smooth muscle cells within the ascending aorta and arch are derived from the cardiac neural crest, it has been suggested that CoA may be a disorder of neural crest development. Animal studies which knock out genes relating to neural crest development have supported this theory, with disruption of PAX3 and NOTCH signalling in the cardiac neural crest leading to both aortic valve abnormalities and thickening of the aortic wall [27,28].

## 4. Clinical Presentation

In the neonate with a discrete significant CoA and no other cardiac lesions, there may be little evidence of a significant pathology under the time of ductal closure, when lower limb perfusion becomes compromised. It is important to note that even in a neonate with a normal echocardiogram soon after closure of the duct, clinically apparent CoA may develop later on [29]. The classic findings are of radio-femoral delay and significant upper and lower limb blood pressure difference. With the closure of the duct and constriction of the CoA segment, there is a sharp increase in the afterload of the left ventricle leading to pressure overload and circulatory collapse. Meanwhile end organ perfusion distal to the CoA is compromised which can lead to a worsening metabolic state, tachypnea and worsening renal function. For this reason, prenatal diagnosis is important so that appropriate measures can be taken at birth for the duct to be kept open with prostaglandin E1 infusion and close monitoring.

Whereas neonates with a discrete CoA may present this way, those with a less severe narrowing may have a delayed presentation even as far as into late adulthood. These patients will usually develop a collateral circulation, raised blood pressure and compensatory left ventricular hypertrophy, followed by LV failure if left untreated. The prognostic benefit of intervention for CoA later in life is uncertain. Table 1 summarises the varied presentation of this condition depending on age.

## 5. Diagnosis—Cardiovascular Imaging

CoA represents a lifelong condition. Even after an apparently successful repair in infancy, there is a high rate of reoperation, reported to be around 50% by the fifth decade. A decreased life expectancy has been shown in long-term follow-up data of repaired CoA patients when compared to the general population, with a high rate of adverse cardiovascular events [30,31]. Multi-modality imaging plays a central role in the diagnosis and follow-up of CoA, with echocardiography, cardiac magnetic resonance (CMR) imaging and cardiac computed tomography (CT) data being integrated for individual patients to aid decision-making for intervention and reintervention throughout their lifetime.

## 6. Fetal Echocardiography

Prenatal diagnosis of CoA is notoriously challenging, with a high rate of false positives on pulse oximetry and fetal echocardiography-based screening, leading to considerable anxiety for families and allocation of limited paediatric and neonatal resources to the admission and surveillance of neonates with equivocal findings. False positive rates range from 48 to 94% [32,33,34]. There are a number of familial, maternal and fetal indications for a comprehensive fetal echocardiography to be undertaken with the majority of studies taking place in the mid-second trimester when the heart is large enough to be visualised clearly, and prior to ossification of the ribs which causes acoustic shadowing. A comprehensive fetal echocardiographic data set is important in raising the suspicion of CoA [35]. The majority of true CoA cases are missed prenatally, and therefore clinicians rightly have a low threshold for enhancing surveillance of those with a suspicion of this diagnosis [36]. For the diagnosis to be made prenatally is highly desirable, since it allows appropriate planning of postnatal care with the use of prostaglandin infusion to prevent closure of the ductus arteriosus, a better preoperative status for the neonate and careful timing of surgery. Prenatal diagnosis of CoA is strongly associated with better neonatal outcomes [37,38].

Studies analysing the performance of fetal echocardiography in the prenatal diagnosis of CoA have found that multiparametric models have a superior diagnostic performance to any one quantitative echocardiographic measure. These models rely on a variety of measurements including those of the LV inflow, as well as measurements comparing the relative ratios of various components of the right and left heart outflows to predict the presence of true CoA. Due to the heterogeneity of the quantitative parameters used, it has been difficult to compare the performance of these models in a meaningful way or integrate these, and none have been adopted into widespread clinical practice. Qualitative data such as the presence of a CoA shelf or hypoplastic arch have also been shown to be predictive of a true diagnosis, though the definition of neonatal aortic arch hypoplasia in itself remains heterogeneous [39]. 

## 7. Paediatric and Adult Echocardiography

**Figure 1:**
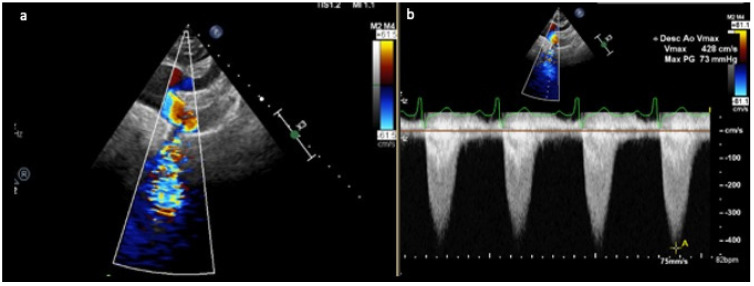
(**a**) Suprasternal view demonstrating a focal area of narrowing of the thoracic aorta distal to the origin of the left subclavian artery with associated flow turbulence on colour flow Doppler. (**b**) Continuous wave Doppler shows increased velocity across the site of coarctation.

Transthoracic echocardiography (TTE) remains the first line of investigation for diagnosis and follow-up of children and adults with CoA. The suprasternal notch view allows visualisation of the aortic anatomy and measurement of Doppler-based gradients across the site of the CoA (Figure 1). Colour flow Doppler allows visualisation of turbulence in association with the site of the obstruction, with pulsed and continuous wave Doppler allowing the peak velocity and flow characteristics to be measured in the descending aorta with a diastolic run-off or ‘tail’ being characteristic of CoA. Doppler assessment of the abdominal aorta is also an important aspect, with a lack of pulsatility associated with severe CoA. TTE also allows a comprehensive assessment of LV function, LV mass and associated cardiac anomalies such as a bicuspid aortic valve. An important aspect of the anatomical assessment is to detect hypoplasia of the aorta, since this will greatly affect the surgical technique employed for repair, with systematic analysis of each segment of the arch in relation to age-matched Z scores [40,41,42].

Limitations of this modality include its operator dependency, as well as the variability in echocardiographic windows between patients which can preclude its use. There is also limited visualisation of extracardiac structures. Transoesophageal echo (TOE) has relatively limited clinical use in the context of CoA due to its comparatively invasive nature and limited visualisation of the aortic arch. 

## 8. Cardiac Computed Tomography

Cardiac computed tomography (CT) has the highest resolution of the non-invasive imaging modalities and allows detailed assessment of the cardiac and non-cardiac structures, including the entire aorta and its branches, collaterals, vascular and extracardiac structures to be ascertained within one examination. Short scanning times allow its use in the paediatric population without the need for sedation, with the possibility of acquiring a complete data set in one or several cardiac cycles, without the need for breath holding. CT does not suffer from the problem of artefacts where a CoA site has been stented.

**Figure 2:**
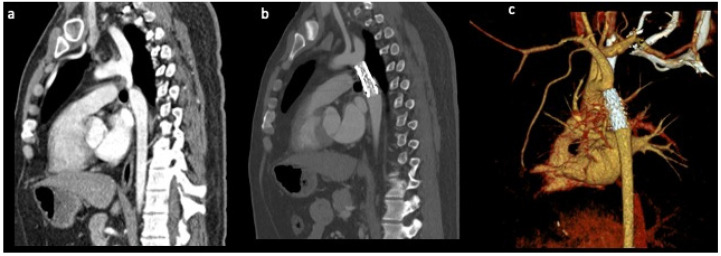
Computed tomography angiography of the aorta in the sagittal planes demonstrating (**a**) the severity of the coarctation (**b**) and after successful stenting. (**c**) Three-dimensional reconstruction displaying the luminal surface of the Cheatham-Platinum covered stent.

Cardiac computed tomography (CT) has the highest spatial resolution of the non-invasive imaging modalities and allows detailed assessment of the cardiac and non-cardiac structures, including the entire aorta and its branches, collateral mapping, surrounding vascular and extracardiac structures to be ascertained within one examination. Short scanning times facilitate its use in the paediatric population, with a short duration of light or milk sedation and the possibility of acquiring a complete data set in one or few cardiac cycles, and overcome the need for breath holding. New reconstruction software and kernels significantly reduce artefacts where a CoA site has been stented (Figure 2). 

An important drawback of cardiac CT is the radiation dose, which is an important factor to consider in a paediatric population destined for lifetime follow-up and repeat cross-sectional imaging. However with the introduction of the multi and new-detector (for instance; photon counting) CT with the recent acquisition techniques, the radiation dose associated with cardiac CT has markedly reduced in the last 20 years. Another important limitation of CT is the lack of haemodynamic assessment, which forms a crucial part of the follow-up of these patients. 

## 9. Cardiac Magnetic Resonance Imaging

**Figure 3:**
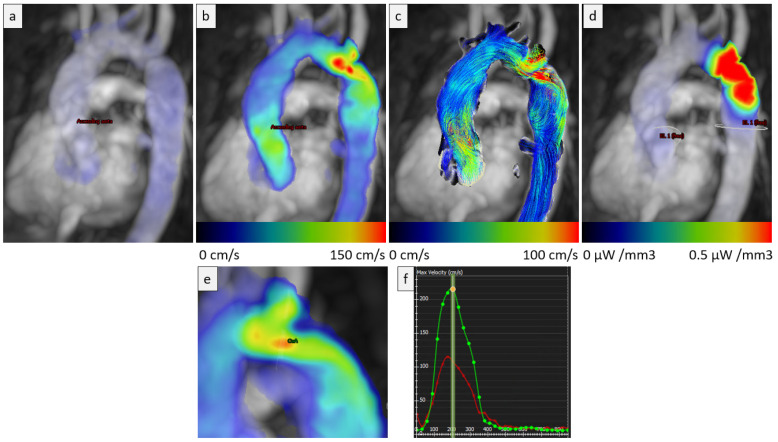
(**a**) Three-dimensional (3D) magnitude image demonstrating coarctation. (**b**) Three-dimensional velocity visual assessment shows flow acceleration in the coarctation. (**c**) Flow streamline visual assessment shows turbulent flow pre and post-stenotic lesion. (**d**) Significant energy dissipation due to the stenotic lesion is mapped by energy loss assessment of 4D flow velocity encoded data. (**e**,**f**) Quantification of peak velocity in 3D through the coarctation (green) and ascending aorta (red). Courtesy of Pankaj Garg—University of East Anglia, UK.

Cardiac magnetic resonance (CMR) imaging has a number of advantages compared to the other modalities, being the gold standard in non-invasive assessment of cardiac structure and function as well as providing non-invasive tissue characterisation through late gadolinium (LGE) imaging. CMR plays a crucial role in the contemporary diagnosis and management of patients with congenital heart disease. With the advent of 4D flow CMR, we are able to integrate sophisticated haemodynamic data with detailed anatomical information for the first time, which is likely to improve our understanding of the heterogenous clinical course seen in many of the patients we treat, including those with CoA (Figure 3). In the follow-up of the patient with repaired CoA, CMR plays an important role in surveillance of the site of operation for complications such as recoarctation and aortic aneurysms, as well as allowing left ventricular volumes, function and mass to be followed up over time, to assess any adverse hemodynamic consequences of a residual gradient or poorly controlled systemic hypertension.

Fetal CMR studies of CoA have demonstrated the potential of 4D CMR to improve prenatal diagnosis of CoA, a notoriously challenging diagnostic area. The importance of flow analysis in CoA may be predicted given the hypothesis that CoA may be a consequence of abnormal or reduced left ventricular flow in fetal life. A recent study by Lloyd et al. demonstrated the superior performance of a multivariate model derived from fetal 4D flow CMR in the third trimester compared to that reported for fetal echocardiographic screening [33]. In a multivariate logistic regression model, the isthmal displacement: descending aortic ratio and the ratio of ascending aorta flow and isthmal flow were the only two significant parameters, with a model incorporating these parameters correctly classifying 95% of true CoA cases and 91% of false positive cases. 

CMR has also been utilised in the research setting to refine our understanding of operated aortic anatomy and haemodynamics, including the impact of aortic arch shape [43] on wall shear stress [44] and subsequent downstream secondary flow patterns. [45] Individual geometry has been shown to have consequences in terms of the incidence of later complications, with a correlation between a ‘gothic’ arch, characterised by a more angulated arch with an increased height-to-width ratio, and resting and exercise-induced hypertension, as well as increased aortic wall stiffness. [46,47,48] Bruse et al. utilised 3D printed models of individual aortic arches in patients with repaired CoA, utilising a novel shape analysis method to derive 3D shape characteristics most correlated to left ventricular ejection fraction and left ventricular diastolic volume. Distinct arch features including characteristics associated with a gothic arch correlated to these indices of cardiac function, a finding which has been replicated in other studies. [49] Flow characteristics of CMR-derived aortic geometries have also been studied through computer simulation to assess the impact of arch shape on flow characteristics, with repaired CoAs demonstrating higher wall shear stress in the descending aorta compared to normal aortas, even where no residual stenosis is present [50]. Thus, we are likely at the beginning of being able to phenotype this patient population in a far more detailed way than previously, with important implications for improving risk stratification and a more personalised approach to treating individual patients with unique anatomical characteristics.

## 10. Diagnosis—Cardiac Catheterisation

Cardiac catheterisation is the gold standard for haemodynamic assessment of CoA and allows high-resolution visualisation of the anatomy of the aortic arch, as well as assessment of any collaterals. A peak-to-peak gradient of >20 mmHg on catheterisation is considered to be evidence of significant CoA in international guidelines [5,6]. The strongest recommendation for intervention is for hypertensive patients with an increased non-invasive gradient between upper and lower limbs, confirmed with invasive measurement (peak-to-peak > 20 mmHg) with a preference for catheter treatment when technically feasible. Table 2 summarises the indications for intervention in CoA.

The role of diagnostic catheterisation is increasingly being superseded by cross-sectional imaging in the surveillance of patients following surgical repair; however, it retains importance where catheter-based intervention for re-stenosis is being considered, whether this is balloon angioplasty or stenting.

## 11. Prognosis 

Untreated CoA has a dismal prognosis, with a 50% mortality rate by age 30 reported in Maude Abbot’s series of 200 patients with unrepaired CoA. Death occurred due to serious sequelae such as aortic rupture, endocarditis and heart failure [51]. Surgical repair is preferred as early as possible after diagnosis, with later surgery being more complex due to the development of a collateral circulation [6].

## 12. Treatment

### Surgery

A number of surgical techniques have been employed for the repair of CoA. In general, the approach is via a left postero-lateral thoracotomy. A reasonably low mortality and morbidity rate from surgery is reported in contemporary studies with 0.54% 30-day mortality reported in a recent study [52]. Recognised complications include left recurrent laryngeal nerve injury, bronchial compression, early recoarctation, persistent hypertension and heart failure. 

**Figure 4:**
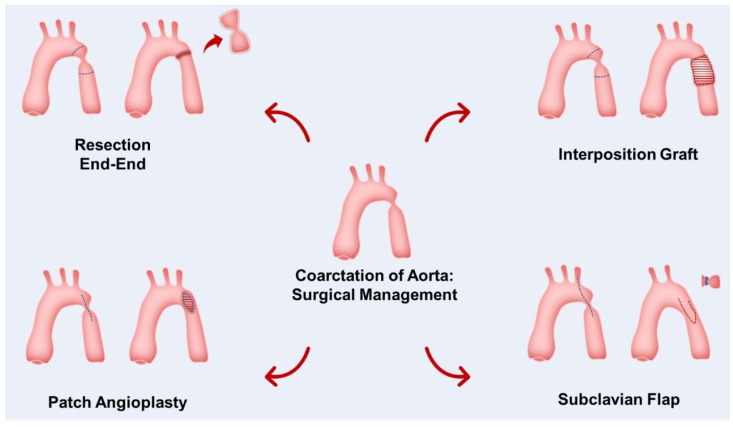
Surgical techniques used for repair of CoA.

Techniques used include end-to-end anastomosis, which involves complete resection of the CoA segment with anastomosis of the two ends of the aorta. Subclavian flap repair (SF) is where the subclavian artery is resected including the CoA segment, with the flap then reversed and used to repair the aorta. Extended end-to-end repair is employed where there is coexistence of a hypoplastic aortic arch and an interposition graft may be used. Patch augmentation is no longer in use, being associated with a high incidence of aortic aneurysms (Figure 4) [53].

Studies comparing these surgical techniques are heterogeneous in a number of aspects, and therefore the superiority of one surgical technique over another remains in doubt in terms of recoarctation and reoperation rates. It has not proved possible to combine these studies in a meta-analysis, with variable hemodynamic definitions of CoA and recoarctation being employed across the literature. In addition, a number of these cohorts are mixed with different proportions of patients with additional congenital diagnoses, ranging from hypoplastic aortic arch and VSD to more complex congenital conditions such as hypoplastic left heart syndrome. There are a number of observational studies that have attempted to answer the question regarding the best surgical technique through a comparison of long-term outcomes following CoA surgery. These show a range of conflicting results, with the majority showing no difference between various techniques [54,55]. There have been calls for a randomised controlled trial to answer this question in a more robust way [56]. However decades on, this is yet to materialise. 

## 13. Transcatheter Interventions

Although surgery remains the first line in children diagnosed with CoA, percutaneous intervention may be used as a strategy in high-risk neonates as definitive treatment, or as a bridge to surgery. Balloon angioplasty and stenting are often used in the post-surgical repair setting, for relief of recoarctation. Catheter-based treatment is recommended as the first line in adults diagnosed with CoA or recoarctation in the latest iteration of the European Society of Cardiology Guidelines for Adult Congenital Heart Disease [6]. The latest AHA/ACC guidelines also stipulate that catheter-based stenting can be considered as a first-line therapy for adults diagnosed with significant CoA [5].

### 13.1. Balloon Angioplasty

Where a percutaneous approach is preferable, balloon angioplasty is typically used in infants < 1 year instead of stent implantation, due to the need for a larger sheath for the latter treatment. Early reports of the long-term outcomes post balloon angioplasty reported a high rate of aneurysm formation compared to a surgical strategy. This, combined with the favourable reintervention rates of <10% reported in longitudinal follow-up of post-surgical repair patients, has led to surgery being considered the first line in this population. However, a recent study of 68 infants aged 3–12 months who underwent balloon angioplasty for native CoA showed this to be a safe and effective management strategy, with early procedural success in 88% (defined as a gradient < 20 mmHg), with 81% freedom from reintervention at 10 years post-procedure. 

### 13.2. Stent Implantation

**Figure 5:**
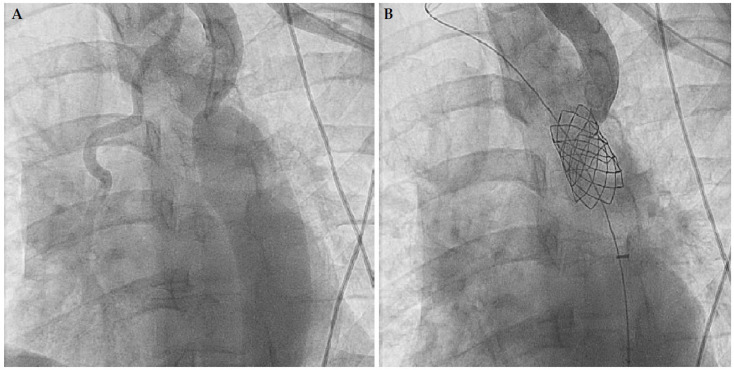
(**A**) Severe coarctation with collaterals and gradient of 30 mmHg across the coarctation segment in a 46-year-old adult male with hypertension, initial diagnosis on echocardiography (**B**) Treated with 45 mm CP stent with final stent diameter of 20 mm and complete resolution of coarctation gradient.

Endovascular stenting has been used preferentially to balloon angioplasty in children > 10 kg due to superior hemodynamic results, lower risk of aneurysm formation, aortic wall injury and restenosis (Figure 5) [57,58]. Bare metal stents and covered stents are both used for this, with the latter having a theoretically lower risk of immediate and late aortic complications, though data on this are limited and mixed [59,60]. The COAST and COAST II trials [61,62] prospectively evaluated Cheatham-Platinum (CP) stents in the treatment of CoA and reported excellent haemodynamic results with a low complication rate in the early follow-up period. However, late follow-up data at 48–60 months reveal a high rate of reintervention (21%), aneurysm formation in both covered and bare CP stent groups (6.3%) and stent fracture (24.4%). [60] It is likely the incidence of aneurysm formation was a significant underestimate, given only 16% of the original cohort had cross-sectional imaging availability at the time of follow-up. The long-term sequelae of stent fracture and aneurysm formation are unknown. 

The role of stenting in smaller children weighing <20 kg has also been studied [63]. Though the numbers reported are small, this appears to be an effective treatment in terms of clinical success, though it is associated with a risk of vascular injury and a high reintervention rate to re-expand the stent at subsequent follow-up. Low-profile pre-mounted stents which are dilatable to adult size are now available and are likely to increase the safety of this procedure from a vascular perspective [64].

## 14. Long-Term Complications

Longitudinal data on repaired CoA reveals a plethora of late complications in spite of contemporary surgical practice and close follow-up. Those who have had a CoA repair continue to have a reduced life expectancy compared to age-matched counterparts in the general population, with only 66% of patients in a recently reported cohort surviving to age 70 [30]. Mortality is generally driven by cardiovascular events, including heart failure and aortic aneurysm rupture. This highlights the importance of lifelong surveillance for late complications, particularly through the use of ambulatory blood pressure monitoring and interval cross-sectional imaging. Hypertension remains a key risk factor for adverse cardiovascular outcomes.

### 14.1. Recoarctation and Reintervention

Recoarctation rates of 30–59% have been reported, with some studies supporting the notion that end-to-end anastomosis is associated with a lower reintervention rate [30,31,55,65]. Age < 1 year at time of repair is associated with a higher rate of reintervention [55]. Aortic valve surgery is required in a proportion of patients due to the known association of CoA with bicuspid aortic valve disease, with ascending aorta surgery being relatively less common [30]. Reintervention rates vary between studies, likely due to the heterogeneity of study populations with respect to the surgical eras included. For example, a study looking at a more contemporary population, who had surgery between 1993 and 2003 reported a much lower reintervention rate of 11% than that seen in cohorts with older patients which typically report a reintervention rate of 20–25% at 30 years [30,31]. A requirement for reintervention is seen more commonly in those who have had a percutaneous intervention or patch aortoplasty.

### 14.2. Hypertension

Hypertension is reported in 25–62% of patients post CoA repair, with the prevalence varying between studies based on the methodology used to define hypertension, with some studies employing 24 h ambulatory BP readings in addition to office or home readings [66]. Risk factors for developing hypertension include older age at time of CoA repair and obesity. The type of intervention or repair does not seem to affect the likelihood of hypertension or significant vascular dysfunction developing [67,68]. Patients with CoA repair display enhanced arterial stiffness and abnormal response to flow even after complete relief of obstruction, lending support to the idea that a generalised central vasculopathy co-exists with the CoA itself [69,70]. Older age at time of initial repair is associated with a greater degree of vascular dysfunction [64,71]. Ambulatory blood pressure readings have been highlighted as approximating most closely to central aortic pressure and left ventricular mass index when compared to home BP readings [72]. Many patients without resting hypertension will develop exercise-induced hypertension (EIH). Although current guidelines do not propose treatment of EIH, this does have prognostic significance with a recent study demonstrating an increased risk of cardiovascular events in these patients [73]. Current international guidelines encourage the use of ambulatory blood pressure monitoring [6]. It remains unknown if stricter blood pressure targets which use exercise-based BP targets would more effectively ameliorate the enhanced cardiovascular risk of these patients. 

### 14.3. Aortic Aneurysm

Approximately 14% of those with surgically repaired CoA develop dilatation of the aorta especially at the site of previous repair, typically defined as a repair site—diaphragm ratio of 150% [65]. Approximately 8–11% develop a discrete aneurysm, more common at the site of previous repair rather than the ascending aorta [30]. False aneurysms are relatively rare but can occur late after the initial repair [74]. The coexistence of bicuspid aortic valve (BAV) and CoA is of importance when assessing the likelihood of aortic complications, with the majority of longitudinal studies of CoA repair including mixed cohorts of patients with and without BAV. BAV in itself is associated with a distinct aortopathy characterised by cystic medical necrosis and a higher risk of aneurysm formation. CoA and BAV therefore interact significantly to enhance the risk of aortic aneurysm formation in the ascending aorta, with aneurysmal dilatation of the ascending aorta being as common as CoA site complications in these patients [75,76]. Ascending aorta aneurysms are twice as likely to develop than those with isolated CoA. Patch repair has been shown to be a risk factor for aneurysm formation in a number of studies [65,75].

### 14.4. Stroke and Coronary Artery Disease

A higher risk of ischaemic and haemorrhagic stroke is observed in CoA patients, in particular subarachnoid haemorrhage as a consequence of the rupture of cerebral aneurysms, which are found more commonly, with a prevalence of 10% in a screening study compared to approximately 2% in the general population [77,78,79,80]. A recent MRI study of the cerebral vasculature in CoA patients highlighted the presence of abnormal intracranial vascular characteristics, with a higher flow velocity and lower distensibility in the carotid arteries denoting higher vascular stiffness, as has been found in the peripheral circulation in previous studies [81]. Taken together, this highlights the importance of secondary risk factor modification in these patients. Interestingly, vascular brain injury was not more common in this cohort of patients compared to controls, which may be an effect of the relatively younger age of this cohort compared to those with long-term follow-up.

The role of premature coronary artery disease (CAD) in driving early mortality in repaired CoA has been controversial in the literature. Although CAD is the primary mode of death in older long-term follow-up studies of repaired CoA, the association of CoA with premature coronary artery disease independent of traditional risk factors such as hypertension has not been conclusively demonstrated [82,83,84,84]. Earlier age at coronary revascularisation and myocardial infarction has been shown compared to the general population in a recent population-based study, with patients with CoA undergoing coronary revascularisation 15 years earlier than those without CoA. In the two studies that have specifically addressed the question of independent risk of premature CAD in CoA patients, no such association has been found [84,85].

### 14.5. LV Remodelling and Failure

CoA leads to pressure overload of the left ventricle and adverse left ventricular remodelling. Histologically, this is characterised by myocyte hypertrophy, disarray and fibrosis. Ensuing alteration in myocardial mechanical properties manifests in the form of diastolic and systolic dysfunction, with LV failure being a significant cause of mortality [30]. Even after complete relief of obstruction, CoA is associated with diastolic dysfunction independent of the presence of coronary artery disease [86,87]. LV mass appears to be significantly correlated to the degree of diastolic dysfunction, with a CoA cohort demonstrating a higher LV mass compared to controls, even after controlling for co-existent hypertension and BMI. Even where systolic function is normal as measured by left ventricular ejection fraction, speckle tracking echocardiography has shown reduced global longitudinal strain indicative of subclinical systolic dysfunction [88]. An aortic isthmus ratio < 0.7 has recently been shown to be an important prognostic factor for reverse left ventricular remodelling after intervention for recoarctation. Pulmonary hypertension has also been shown to be an important prognostic factor predictive of cardiovascular events [89]. 

## 15. Pregnancy

Pregnancy is categorised as an intermediate risk in international guidelines in the setting of repaired CoA (modified WHO classification II–III) and high risk in those with unrepaired CoA (mWHO IV) [5,90,91]. This is based largely on consensus opinion, with the majority of studies being retrospective and reporting on relatively small cohorts [92,93]. There are individual reports of maternal mortality due to aortic dissection, which is one of the most feared complications and for which multiple additional risk factors can co-exist, for example in a patient with CoA, bicuspid aortic valve and Turner’s syndrome [94,95]. However, pregnancy appears to be well tolerated, with the majority of contemporary cohort studies reporting no maternal mortality [92,96,97]. One of the largest studies of pregnancy outcomes in CoA to date is based on the worldwide prospective registry of pregnancy and cardiac disease (ROPAC) [98]. This revealed a lower risk profile than would be suggested by the mWHO classification, reporting on 303 pregnancies with no maternal mortality. The low major adverse cardiovascular event (MACE) rate of 4.3% was driven by heart failure admissions in 3.3% in the second and third trimesters. Interestingly, there was no evidence of an increased risk of hypertensive disorders of pregnancy or pre-eclampsia compared to the general population. This is in contrast to the majority of published data which suggest a significantly elevated risk of hypertension in pregnancy [92,93]. The high rate of pre-existing cardiac medication use (41%) suggests that this was a cohort with relatively good provision of ongoing cardiac care and possible selection bias. Risk factors for MACE included pre-existing heart failure and pre-existing cardiac medication use, as well as origin from an emerging country. In the 29 patients with unrepaired CoA, there was a high rate of Caesarean section (71%) but no evidence of an increase in adverse maternal or fetal outcomes compared to those with repaired CoA. Although these data are reassuring, as with most registry data, little is known regarding the disease severity in these patients with no haemodynamic data provided. However, it does seem that in the context of an asymptomatic patient with repaired CoA and preserved left ventricular function, pregnancy is relatively lower risk than was previously thought.

## 16. Conclusions

CoA remains a diagnostic and therapeutic challenge due to the difficulties of prenatal diagnosis, wide-ranging anatomical variation, long-term adverse cardiovascular sequelae of abnormal haemodynamics and impaired systemic vascular function, which are still not fully understood. Even where there is timely repair, these patients remain at enhanced cardiovascular risk throughout their lifetime. In the adult population, it is important to regularly screen for hypertension as well as repair site-related complications with interval surveillance cross-sectional imaging. The increasing use of advanced imaging techniques such as 4D flow MRI, which is able to noninvasively combine anatomical and haemodynamic data, is likely to refine the prognostication of these patients which is sorely needed. The reduced life expectancy of these patients highlights a need to more accurately phenotype this heterogenous patient population to help better understand the pathophysiological factors behind enhanced cardiovascular risk, despite successful repair, and the best indications and optimal timing for reintervention.

## Figures and Tables

**Table 1:** Summary of the spectrum of different clinical presentations of Coarctation of the Aorta.

Age Group	Clinical Symptoms	Examination and Investigation Findings
Neonates—early presentation	Often asymptomatic prior to ductal closure Tachypnoea Difficulty feeding/failure to thrive	Pulse oximetry-based screening may reveal reduced saturations in the lower limbs compared to the upper limbs, due to left to right shunting through the patent ductus arteriosus Diminished femoral pulses Oliguria, renal failure Left ventricular failure and cardiogenic shock Echo: narrowed aortic segment with pressure gradient across it, ductal patency with left to right shunt Signs of any associated congenital heart lesions, e.g., VSD or aortic stenosis.
Neonates— late presentation	Tachypnoea Difficult feeding/failure to thrive	Diminished femoral pulses Intrascapular systolic murmur Echo: Left ventricular hypertrophy and left ventricular failure in addition to CoA ECG reveals left ventricular hypertrophy. CXR may show cardiomegaly and signs of interstitial pulmonary oedema
Children–Adults	Often asymptomatic Headaches, epistaxis Lower limb claudication Reduced exercise capacity May more rarely present with symptoms related to complications due to long-term undiagnosed hypertension including coronary artery disease and heart failure	Uncontrolled hypertension Upper and lower limb BP discrepancy Continuous murmur due to collaterals Echo: left ventricular hypertrophy, left ventricular failure, May more rarely present with catastrophic sequelae such as aortic dissection or intracranial haemorrhage

**Table 2:** Indications for treatment of coarctation and re-coarctation of the aorta, adapted from 2020 European Society of Cardiology Adult Congenital Heart Disease guidelines [6].

Indications for Treatment
Surgical or catheter-based treatment indicated in hypertensive patients with an increased non-invasive gradient between upper and lower limbs confirmed with invasive measurement (peak to peak > 20 mmHg) with preference for catheter treatment (stenting)
Catheter treatment should be considered in hypertensive patients with >50% narrowing relative to the aortic diameter at the diaphragm, even if the invasive peak-to-peak gradient is <20 mmHg
Catheter treatment should be considered in normotensive patients with an increased non-invasive gradient confirmed with invasive measurement (peak-to-peak > 20 mmhg)
Catheter treatment may be considered in normotensive patients with >50% narrowing relative to the aortic diameter at the diaphragm, even if the invasive peak-to-peak gradient is <20 mmHg

## Data Availability

No new data were created or analyzed in this study. Data sharing is not applicable to this article.

## References

[1] Roger V.L., Go A.S., Lloyd-Jones D.M., Adams R.J., Berry J.D., Brown T.M., Carnethon M.R., Dai S., De Simone G., Ford E.S. (2011). Heart Disease and Stroke Statistics—2011 Update. A Report from the American Heart Association. Circulation.

[2] Reller M.D., Strickland M.J., Riehle-Colarusso T., Mahle W.T., Correa A. (2008). Prevalence of Congenital Heart Defects in Metropolitan Atlanta, 1998–2005. J. Pediatr..

[3] Crafoord C., Nylin G. (1945). Congenital coarctation of the aorta and its surgical treatment. J. Thorac. Surg..

[4] Hoschtitzky J.A., Anderson R.H., Elliott M.J., Anderson R.H., Baker E.J., Penny D.J., Redington A.N., Rigby M.L., Wernovsky G. (2010). CHAPTER 46—Aortic Coarctation and Interrupted Aortic Arch. Paediatric Cardiology.

[5] Stout K.K., Daniels C.J., Aboulhosn J.A., Bozkurt B., Broberg C.S., Colman J.M., Crumb S.R., Dearani J.A., Fuller S., Gurvitz M. (2019). 2018 AHA/ACC Guideline for the Management of Adults with Congenital Heart Disease: A Report of the American College of Cardiology/American Heart Association Task Force on Clinical Practice Guidelines. Circulation.

[6] Baumgartner H., De Backer J., Babu-Narayan S.V., Budts W., Chessa M., Diller G.-P., Lung B., Kluin J., Lang I.M., Meijboom F. (2021). 2020 ESC Guidelines for the Management of Adult Congenital Heart Disease: The Task Force for the Management of Adult Congenital Heart Disease of the European Society of Cardiology (ESC). Endorsed by: Association for European Paediatric and Congenital Card. Eur. Heart J..

[7] Keshavarz-Motamed Z., Edelman E.R., Motamed P.K., Garcia J., Dahdah N., Kadem L. (2015). The role of aortic compliance in determination of coarctation severity: Lumped parameter modeling, in vitro study and clinical evaluation. J. Biomech..

[8] Teo L.L.S., Cannell T., Babu-Narayan S.V., Hughes M., Mohiaddin R.H. (2011). Prevalence of Associated Cardiovascular Abnormalities in 500 Patients with Aortic Coarctation Referred for Cardiovascular Magnetic Resonance Imaging to a Tertiary Center. Pediatr. Cardiol..

[9] Becker A.E., Becker M.J., Edwards J.E. (1970). Anomalies Associated with Coarctation of Aorta Particular Reference to Infancy. Circulation.

[10] Grattan M., Prince A., Rumman R.K., Morgan C., Petrovic M., Hauck A., Young L., Franco-Cereceda A., Loeys B., Mohamed S.A. (2020). Predictors of Bicuspid Aortic Valve–Associated Aortopathy in Childhood. Circ. Cardiovasc. Imaging.

[11] Shone J.D., Sellers R.D., Anderson R.C., Adams P., Lillehei C., Edwards J.E. (1963). The developmental complex of “parachute mitral valve”, supravalvular ring of left atrium, subaortic stenosis, and coarctation of aorta. Am. J. Cardiol..

[12] Aslam S., Khairy P., Shohoudi A., Mercier L.-A., Dore A., Marcotte F., Miró J., Avila-Alonso P., Ibrahim R., Asgar A. (2017). Shone Complex: An Under-recognized Congenital Heart Disease with Substantial Morbidity in Adulthood. Can. J. Cardiol..

[13] Warnes C.A. (2003). Bicuspid aortic valve and coarctation: Two villains part of a diffuse problem. Heart.

[14] Ellesøe S.G., Workman C.T., Bouvagnet P., Loffredo C.A., McBride K.L., Hinton R.B., van Engelen K., Gertsen E.C., Mulder B.J.M., Postma A.V. (2018). Familial co-occurrence of congenital heart defects follows distinct patterns. Eur. Heart J..

[15] Parker L.E., Landstrom A.P. (2021). Genetic Etiology of Left-Sided Obstructive Heart Lesions: A Story in Development. J. Am. Heart Assoc..

[16] Garg V., Muth A.N., Ransom J.F., Schluterman M.K., Barnes R., King I.N., Grossfeld P.D., Srivastava D. (2005). Mutations in NOTCH1 cause aortic valve disease. Nature.

[17] Andelfinger G., Tapper A.R., Welch R.C., Vanoye C.G., George A.L., Benson D.W. (2002). KCNJ2 Mutation Results in Andersen Syndrome with Sex-Specific Cardiac and Skeletal Muscle Phenotypes. Am. J. Hum. Genet..

[18] Prandstraller D., Mazzanti L., Picchio F., Magnani C., Bergamaschi R., Perri A., Tsingos E., Cacciari E. (1999). Turner’s Syndrome: Cardiologic Profile According to the Different Chromosomal Patterns and Long-Term Clinical Follow-Up of 136 Nonpreselected Patients. Pediatr. Cardiol..

[19] Krag-Olsen B., Nielsen J., Sorensen B.K.E. (1994). Prevalence of Cardiovascular Malformations and Association with Karyotypes in Turner’s Syndrome. Arch. Dis. Child..

[20] Yokoyama U., Ichikawa Y., Minamisawa S., Ishikawa Y. (2017). Pathology and molecular mechanisms of coarctation of the aorta and its association with the ductus arteriosus. J. Physiol. Sci..

[21] Kim H.S., Aikawa M., Kimura K., Kuro-o M., Nakahara K., Suzuki T., Katoh H., Okamoto E., Yazaki Y., Nagai R. (1993). Ductus arteriosus. Advanced differentiation of smooth muscle cells demonstrated by myosin heavy chain isoform expression in rabbits. Circulation.

[22] Slomp J., Gittenberger-de Groot A.C., Glukhova M.A., van Munsteren J.C., Kockx M.M., Schwartz S.M., Koteliansky V.E. (1997). Differentiation, Dedifferentiation, and Apoptosis of Smooth Muscle Cells during the Development of the Human Ductus Arteriosus. Arterioscler. Thromb. Vasc. Biol..

[23] Elzenga N.J., Groot A.C.G.-D. (1983). Localised coarctation of the aorta. An age dependent spectrum. Br. Heart J..

[24] Jimenez M., Daret D., Choussat A., Bonnet J. (1999). Immunohistological and ultrastructural analysis of the intimal thickening in coarctation of human aorta. Cardiovasc. Res..

[25] Anaskovic I.R.T., Lic S.L.I., Urisic V.L.J., Ackovic M.I.L., Ilosavljevic Z.O.M. (2019). Histochemical, Immunohisto-chemical and Ultrastructural. Rom. J. Morphol. Embryol..

[26] Jonas R.A. (1991). Coarctation: Do we need to resect ductal tissue?. Ann. Thorac. Surg..

[27] High F.A., Zhang M., Proweller A., Tu L., Parmacek M.S., Pear W.S., Epstein J.A. (2007). An essential role for Notch in neural crest during cardiovascular development and smooth muscle differentiation. J. Clin. Investig..

[28] Jain R., Engleka K.A., Rentschler S.L., Manderfield L.J., Li L., Yuan L., Epstein J.A. (2011). Cardiac neural crest orchestrates remodeling and functional maturation of mouse semilunar valves. J. Clin. Investig..

[29] Head C.E.G., Jowett V.C., Sharland G.K., Simpson J.M. (2005). Timing of presentation and postnatal outcome of infants suspected of having coarctation of the aorta during fetal life. Heart.

[30] Lee M.G.Y., Babu-Narayan S.V., Kempny A., Uebing A., Montanaro C., Shore D.F., D’udekem Y., Gatzoulis M.A. (2019). Long-term mortality and cardiovascular burden for adult survivors of coarctation of the aorta. Heart.

[31] Choudhary P., Canniffe C., Jackson D.J., Tanous D., Walsh K., Celermajer D.S. (2015). Late outcomes in adults with coarctation of the aorta. Heart.

[32] Hede S.V., DeVore G., Satou G., Sklansky M. (2020). Neonatal management of prenatally suspected coarctation of the aorta. Prenat. Diagn..

[33] Lloyd D.F., van Poppel M.P., Pushparajah K., Vigneswaran T.V., Zidere V., Steinweg J., van Amerom J.F., Roberts T.A., Schulz A., Charakida M. (2021). Analysis of 3-Dimensional Arch Anatomy, Vascular Flow, and Postnatal Outcome in Cases of Suspected Coarctation of the Aorta Using Fetal Cardiac Magnetic Resonance Imaging. Circ. Cardiovasc. Imaging.

[34] Gómez-Montes E., Herraiz I., Mendoza A., Escribano D., Galindo A. (2013). Prediction of coarctation of the aorta in the second half of pregnancy. Ultrasound Obstet. Gynecol..

[35] Quaresima P., Fesslova V., Farina A., Kagan K.O., Candiani M., Morelli M., Crispi F., Cavoretto P.I. (2023). How to Do a Fetal Cardiac Scan. Archives of Gynecology and Obstetrics.

[36] Quartermain M.D., Hill K.D., Goldberg D.J., Jacobs J.P., Jacobs M.L., Pasquali S.K., Verghese G.R., Wallace A.S., Ungerleider R.M. (2019). Prenatal Diagnosis Influences Preoperative Status in Neonates with Congenital Heart Disease: An Analysis of the Society of Thoracic Surgeons Congenital Heart Surgery Database. Pediatr. Cardiol..

[37] Franklin O., Burch M., Manning N., Sleeman K., Gould S., Archer N. (2002). Prenatal diagnosis of coarctation of the aorta improves survival and reduces morbidity. Heart.

[38] Eapen R.S., Rowland D.G., Franklin W.H. (1998). Effect of Prenatal Diagnosis of Critical Left Heart Obstruction on Perinatal Morbidity and Mortality. Am. J. Perinatol..

[39] Langley S.M., Sunstrom R.E., Reed R.D., Rekito A.J., Gerrah R. (2013). The Neonatal Hypoplastic Aortic Arch: Decisions and More Decisions. Semin. Thorac. Cardiovasc. Surg. Pediatr. Card. Surg. Annu..

[40] Goudar S.P., Shah S.S., Shirali G.S. (2016). Echocardiography of Coarctation of the Aorta, Aortic Arch Hypoplasia, and Arch Interruption: Strategies for Evaluation of the Aortic Arch. Cardiol. Young.

[41] Şişli E., Kalın S., Tuncer O.N., Ayık M.F., Alper H., Levent R.E., Şahin H., Atay Y. (2019). Comparison Between Nomograms Used to Define Pediatric Aortic Arch Hypoplasia: Retrospective Evaluation among Patients Less Than 1 Year Old with Coarctation of the Aorta. Pediatr. Cardiol..

[42] Pettersen M.D., Du W., Skeens M.E., Humes R.A. (2008). Regression Equations for Calculation of Z Scores of Cardiac Structures in a Large Cohort of Healthy Infants, Children, and Adolescents: An Echocardiographic Study. J. Am. Soc. Echocardiogr..

[43] Bruse J.L., Khushnood A., McLeod K., Biglino G., Sermesant M., Pennec X., Taylor A.M., Hsia T.-Y., Schievano S., Khambadkone S. (2017). How successful is successful? Aortic arch shape after successful aortic coarctation repair correlates with left ventricular function. J. Thorac. Cardiovasc. Surg..

[44] Van Ooij P., Farag E.S., Blanken C.P.S., Nederveen A.J., Groenink M., Planken R.N., Boekholdt S.M. (2021). Fully quantitative mapping of abnormal aortic velocity and wall shear stress direction in patients with bicuspid aortic valves and repaired coarctation using 4D flow cardiovascular magnetic resonance. J. Cardiovasc. Magn. Reson..

[45] Zhang Y., Zhang R., Thomas N., Ullah A.H., Eichholz B., Estevadeordal J., Suzen Y.B. (2022). Experimental and computational study of pulsatile flow characteristics in Romanesque and gothic aortic arch models. Med. Eng. Phys..

[46] Ou P., Bonnet D., Auriacombe L., Pedroni E., Balleux F., Sidi D., Mousseaux E. (2004). Late systemic hypertension and aortic arch geometry after successful repair of coarctation of the aorta. Eur. Heart J..

[47] Donazzan L., Crepaz R., Stuefer J., Stellin G. (2014). Abnormalities of Aortic Arch Shape, Central Aortic Flow Dynamics, and Distensibility Predispose to Hypertension after Successful Repair of Aortic Coarctation. World J. Pediatr. Congenit. Heart Surg..

[48] Ou P., Celermajer D.S., Mousseaux E., Giron A., Aggoun Y., Szezepanski I., Sidi D., Bonnet D. (2007). Vascular Remodeling after “Successful” Repair of Coarctation. J. Am. Coll. Cardiol..

[49] Sophocleous F., Biffi B., Milano E.G., Bruse J., Caputo M., Rajakaruna C., Schievano S., Emanueli C., Bucciarelli-Ducci C., Biglino G. (2019). Aortic morphological variability in patients with bicuspid aortic valve and aortic coarctation. Eur. J. Cardio-Thoracic Surg..

[50] Goodarzi Ardakani V., Goordoyal H., Ordonez M.V., Sophocleous F., Curtis S., Bedair R., Caputo M., Gambaruto A., Biglino G. (2022). Isolating the Effect of Arch Architecture on Aortic Hemodynamics Late after Coarctation Repair: A Computational Study. Front. Cardiovasc. Med..

[51] Abbott M.E. (1928). Coarctation of the aorta of the adult type: II. A statistical study and historical retrospect of 200 recorded cases with autopsy, of stenosis or obliteration of the descending arch in subjects above the age of two years. Am. Heart J..

[52] Tulzer A., Mair R., Kreuzer M., Tulzer G. (2016). Outcome of aortic arch reconstruction in infants with coarctation: Importance of operative approach. J. Thorac. Cardiovasc. Surg..

[53] Walhout R.J., Lekkerkerker J.C., Oron G.H., Hitchcock F.J., Meijboom E.J., Bennink G.B. (2003). Comparison of polytetrafluoroethylene patch aortoplasty and end-to-end anastomosis for coarctation of the aorta. J. Thorac. Cardiovasc. Surg..

[54] Mery C.M., Guzmán-Pruneda F.A., Trost J.G., McLaughlin E., Smith B.M., Parekh D.R., Adachi I., Heinle J.S., McKenzie E.D., Fraser C.D. (2015). Contemporary Results of Aortic Coarctation Repair Through Left Thoracotomy. Ann. Thorac. Surg..

[55] Brown M.L., Burkhart H.M., Connolly H.M., Dearani J.A., Cetta F., Li Z., Oliver W.C., Warnes C.A., Schaff H.V. (2013). Coarctation of the Aorta: Lifelong Surveillance Is Mandatory Following Surgical Repair. J. Am. Coll. Cardiol..

[56] Jahangiri M., Shinebourne E.A., Zurakowski D., Rigby M.L., Redington A.N., Lincoln C. (2000). Subclavian flap angioplasty: Does the arch look after itself?. J. Thorac. Cardiovasc. Surg..

[57] Forbes T.J., Kim D.W., Du W., Turner D.R., Holzer R., Amin Z., Hijazi Z., Ghasemi A., Rome J.J., Nykanen D. (2011). Comparison of Surgical, Stent, and Balloon Angioplasty Treatment of Native Coarctation of the Aorta: An Observational Study by the CCISC (Congenital Cardiovascular Interventional Study Consortium). J. Am. Coll. Cardiol..

[58] Salcher M., Naci H., Law T.J., Kuehne T., Schubert S., Kelm M., Morley-Fletcher E., Hennemuth A., Manset D., Mcguire A. (2016). Balloon Dilatation and Stenting for Aortic Coarctation. Circ. Cardiovasc. Interv..

[59] Butera G., Manica J.L.L., Marini D., Piazza L., Chessa M., Filho R.I.R., Leite R.E.S., Carminati M. (2014). From Bare to Covered. Catheter. Cardiovasc. Interv..

[60] Holzer R.J., Gauvreau K., McEnaney K., Watanabe H., Ringel R. (2021). Long-Term Outcomes of the Coarctation of the Aorta Stent Trials. Circ. Cardiovasc. Interv..

[61] Meadows J., Minahan M., McElhinney D.B., McEnaney K., Ringel R. (2015). Intermediate Outcomes in the Prospective, Multicenter Coarctation of the Aorta Stent Trial (COAST). Circulation.

[62] Taggart N.W., Minahan M., Cabalka A.K., Cetta F., Usmani K., Ringel R.E. (2016). Immediate Outcomes of Covered Stent Placement for Treatment or Prevention of Aortic Wall Injury Associated with Coarctation of the Aorta (COAST II). JACC Cardiovasc. Interv..

[63] Boe B.A., Armstrong A.K., Janse S.A., Loccoh E.C., Stockmaster K., Holzer R.J., Cheatham S.L., Cheatham J.P., Berman D.P. (2021). Percutaneous Implantation of Adult Sized Stents for Coarctation of the Aorta in Children ≤20 kg. Circ. Cardiovasc. Interv..

[64] Kang S.-L., Tometzki A., Taliotis D., Martin R. (2017). Stent Therapy for Aortic Coarctation in Children <30 Kg: Use of the Low Profile Valeo Stent. Pediatr. Cardiol..

[65] Chen S.S.M., Dimopoulos K., Alonso-Gonzalez R., Liodakis E., Teijeira-Fernandez E., Alvarez-Barredo M., Kempny A., Diller G., Uebing A., Shore D. (2014). Prevalence and prognostic implication of restenosis or dilatation at the aortic coarctation repair site assessed by cardiovascular MRI in adult patients late after coarctation repair. Int. J. Cardiol..

[66] Canniffe C., Ou P., Walsh K., Bonnet D., Celermajer D. (2013). Hypertension after repair of aortic coarctation—A systematic review. Int. J. Cardiol..

[67] Martins J.D., Zachariah J., Selamet Tierney E.S., Truong U., Morris S.A., Kutty S., de Ferranti S.D., Guarino M., Thomas B., Oliveira D. (2019). Impact of Treatment Modality on Vascular Function in Coarctation of the Aorta: The LOVE-COARCT Study. J. Am. Heart Assoc..

[68] Pieper T., Latus H., Schranz D., Kreuder J., Reich B., Gummel K., Hudel H., Voges I. (2019). Aortic elasticity after aortic coarctation relief: Comparison of surgical and interventional therapy by cardiovascular magnetic resonance imaging. BMC Cardiovasc. Disord..

[69] De Divitiis M., Pilla C., Kattenhorn M., Zadinello M., Donald A., Leeson P., Wallace S., Redington A., Deanfield J.E. (2001). Vascular Dysfunction after Repair of Coarctation of the Aorta. Circulation.

[70] Gardiner H.M., Celermajer D.S., Sorensen K., Georgakopoulos D., Robinson J., Thomas O., Deanfield J.E. (1994). Arterial reactivity is significantly impaired in normotensive young adults after successful repair of aortic coarctation in childhood. Circulation.

[71] Heger M., Willfort A., Neunteufl T., Rosenhek R., Gabriel H., Wollenek G., Wimmer M., Maurer G., Baumgartner H. (2005). Vascular dysfunction after coarctation repair is related to the age at surgery. Int. J. Cardiol..

[72] Egbe A.C., Miranda W.R., Connolly H.M., Kullo I.J. (2021). Ambulatory blood pressure data is the best approximation of central aortic pressure in coarctation of aorta. Int. J. Cardiol. Congenit. Heart Dis..

[73] Egbe A.C., Miranda W.R., Jain C.C., Borlaug B.A., Connolly H.M. (2022). Prognostic Implications of Exercise-Induced Hypertension in Adults with Repaired Coarctation of Aorta. Hypertension.

[74] Somers T., Nies H.M.J.M., van Kimmenade R.R.J., Bosboom D.G.H., Geuzebroek G.S.C., Morshuis W.J. (2022). Necessity of life-long follow-up after surgery for coarctation of the aorta: A case series of very late false aneurysm formation. Eur. Heart J. Case Rep..

[75] Von Kodolitsch Y., Aydin M.A., Koschyk D.H., Loose R., Schalwat I., Karck M., Cremer J., Haverich A., Berger J., Meinertz T. (2002). Predictors of aneurysmal formation after surgical correction of aortic coarctation. J. Am. Coll. Cardiol..

[76] Lim M.S., Cordina R., Kotchetkova I., Celermajer D.S. (2022). Late complication rates after aortic coarctation repair in patients with or without a bicuspid aortic valve. Heart.

[77] Pickard S.S., Gauvreau K., Gurvitz M., Gagne J.J., Opotowsky A.R., Jenkins K.J., Prakash A. (2023). Stroke in Adults with Coarctation of the Aorta: A National Population-Based Study. J. Am. Heart Assoc..

[78] Giang K.W., Fedchenko M., Dellborg M., Eriksson P., Mandalenakis Z. (2021). Burden of Ischemic Stroke in Patients with Congenital Heart Disease: A Nationwide, Case-Control Study. J. Am. Heart Assoc..

[79] Giang K.W., Mandalenakis Z., Dellborg M., Lappas G., Eriksson P., Hansson P.-O., Rosengren A. (2018). Long-Term Risk of Hemorrhagic Stroke in Young Patients with Congenital Heart Disease. Stroke.

[80] Curtis S., Bradley M., Wilde P., Aw J., Chakrabarti S., Hamilton M., Martin R., Turner M., Stuart A.G. (2012). Results of Screening for Intracranial Aneurysms in Patients with Coarctation of the Aorta. Am. J. Neuroradiol..

[81] Meijs T.A., van Tuijl R.J., van den Brink H., Weaver N.A., Siero J.C.W., van der Worp H.B., Braun K.P.J., Leiner T., de Jong P.A., Zwanenburg J.J.M. (2023). Assessment of aortic and cerebral haemodynamics and vascular brain injury with 3 and 7 T magnetic resonance imaging in patients with aortic coarctation. Eur. Heart J. Open.

[82] Cokkinos D.V., Leachman R.D., Cooley D.A. (1979). Increased mortality rate from coronary artery disease following operation for coarctation of the aorta at a late age. J. Thorac. Cardiovasc. Surg..

[83] Toro-Salazar O.H., Steinberger J., Thomas W., Rocchini A.P., Carpenter B., Moller J.H. (2002). Long-term follow-up of patients after coarctation of the aorta repair. Am. J. Cardiol..

[84] Roifman I., Therrien J., Ionescu-Ittu R., Pilote L., Guo L., Kotowycz M.A., Martucci G., Marelli A.J. (2012). Coarctation of the Aorta and Coronary Artery Disease. Circulation.

[85] Egbe A.C., Rihal C.S., Thomas A., Boler A., Mehra N., Andersen K., Kothapalli S., Taggart N.W., Connolly H.M. (2019). Coronary Artery Disease in Adults with Coarctation of Aorta: Incidence, Risk Factors, and Outcomes. J. Am. Heart Assoc..

[86] Moskowitz W.B., Schieken R.M., Mosteller M., Bossano R. (1990). Altered systolic and diastolic function in children after “successful” repair of coarctation of the aorta. Am. Heart J..

[87] Egbe A.C., Miranda W.R., Connolly H.M. (2020). Increased prevalence of left ventricular diastolic dysfunction in adults with repaired coarctation of aorta. IJC Heart Vasc..

[88] Menting M.E., van Grootel R.W.J., van den Bosch A.E., Eindhoven J.A., McGhie J.S., Cuypers J.A.A.E., Witsenburg M., Helbing W.A., Roos-Hesselink J.W. (2016). Quantitative assessment of systolic left ventricular function with speckle-tracking echocardiography in adult patients with repaired aortic coarctation. Int. J. Cardiovasc. Imaging.

[89] Egbe A.C., Miranda W.R., Jain C.C., Connolly H.M. (2021). Right Heart Dysfunction in Adults with Coarctation of Aorta: Prevalence and Prognostic Implications. Circ. Cardiovasc. Imaging.

[90] Regitz-Zagrosek V., Roos-Hesselink J.W., Bauersachs J., Blomström-Lundqvist C., Cífková R., De Bonis M., Iung B., Johnson M.R., Kintscher U., Kranke P. (2018). 2018 ESC Guidelines for the Management of Cardiovascular Diseases during Pregnancy: The Task Force for the Management of Cardiovascular Diseases during Pregnancy of the European Society of Cardiology (ESC). Eur. Heart. J..

[91] Van Hagen I.M., Roos-Hesselink J.W. (2020). Pregnancy in congenital heart disease: Risk prediction and counselling. Heart.

[92] Vriend J.W.J., Drenthen W., Pieper P.G., Roos-Hesselink J.W., Zwinderman A.H., van Veldhuisen D.J., Mulder B.J.M., on behalf of the ZAHARA Investigators (2005). Outcome of Pregnancy in Patients after Repair of Aortic Coarctation. Eur. Heart J..

[93] Krieger E.V., Landzberg M.J., Economy K.E., Webb G.D., Opotowsky A.R. (2011). Comparison of Risk of Hypertensive Complications of Pregnancy among Women with versus without Coarctation of the Aorta. Am. J. Cardiol..

[94] Beauchesne L.M., Connolly H.M., Ammash N.M., Warnes C.A. (2001). Coarctation of the aorta: Outcome of pregnancy. J. Am. Coll. Cardiol..

[95] Plunkett M.D., Bond L.M., Geiss D.M. (2000). Staged repair of acute Type I aortic dissection and coarctation in pregnancy. Ann. Thorac. Surg..

[96] Siegmund A.S., Kampman M.A.M., Bilardo C.M., Balci A., van Dijk A.P.J., Oudijk M.A., Mulder B.J.M., Roos-Hesselink J.W., Sieswerda G.T., Koenen S.V. (2017). Pregnancy in women with corrected aortic coarctation: Uteroplacental Doppler flow and pregnancy outcome. Int. J. Cardiol..

[97] Saidi A., Bezold L., Altman C., Ayres N., Bricker J.T. (1998). Outcome of pregnancy following intervention for coarctation of the aorta. Am. J. Cardiol..

[98] Ramlakhan K.P., Tobler D., Greutmann M., Schwerzmann M., Baris L., Yetman A.T., Nihoyannopoulos P., Manga P., Boersma E., Maggioni A.P. (2021). Pregnancy outcomes in women with aortic coarctation. Heart.

